# IllumiSIFT: A Cascade Framework for DoG Pyramid Learning in Darkness

**DOI:** 10.3390/s26072147

**Published:** 2026-03-31

**Authors:** Dewan Fahim Noor, Mohammed Rashid Chowdhury, Sadia Sikder

**Affiliations:** 1 Electrical and Computer Engineering Department, Tuskegee University, Tuskegee, AL 36088, USA; 2 CGI Inc., Ottawa, ON K1P 6A4, Canada; rashid0531@gmail.com; 3 Pharmavite LLC, Opelika, AL 36801, USA; sadiasikder692@gmail.com

**Keywords:** deep learning, object recognition, SIFT key points, residual learning, low-light images, DoG images

## Abstract

In visual object recognition problems, low light exposure and low-quality images present significant challenges in navigation, surveillance, and image retrieval applications, where reliable feature detection is critical. Although recent deep learning–based image enhancement methods improve visual quality in the pixel domain, these improvements often do not translate to downstream machine vision performance, as important local gradient structures required for stable key point detection are frequently suppressed. In this work, we propose IllumiSIFT, a task-driven dark image enhancement framework that focuses on preserving Scale-Invariant Feature Transform (SIFT) key points by directly learning the Difference-of-Gaussian (DoG) pyramid from low-light image inputs. Unlike conventional pixel-level recovery approaches, the proposed method employs a cascaded residual learning architecture to predict Gaussian-blurred representations at multiple scales, enabling the generation of enhanced DoG images that are inherently aligned with the SIFT detection process. Extensive experiments conducted on the CDVS, Oxford Buildings, and Paris datasets demonstrate that the proposed approach consistently outperforms state-of-the-art enhancement methods in downstream SIFT matching performance under severe low-light conditions. These results confirm that gradient-domain, task-aligned enhancement provides a more effective and practical solution for recognition-centric low-light imaging applications.

## 1. Introduction

One of the most pertinent challenges in image processing and computer vision is dealing with low-light imaging. With less visibility and low-quality images, the recognition task gets tougher as the detection points become very difficult to be extracted with the loss of contrast and features in images. Modern-day image sensors have to deal with low peak signal to noise ratios (PSNR) as the low-light environment results in producing very noisy images. Moreover, the depth of the field gets reduced if the aperture is enlarged. This makes the images blurry. However, if the exposure time is lengthened, it will again cause motion blur and corrupt the images. These challenges force us to invest time in enhancing low-light images by reproducing scenes with correct exposure. However, while enhancing the images by aiming to reduce the PSNR, we might lose the key feature points of the images which are fundamental in recognition problems. So, enhancing the key feature points from low-light images proves to be an instrumental solution in this kind of image recognition problem.

There have been extensive studies on the improvement of low-light images with noisy short exposure. In [1], the image denoising algorithm BM3D was used to enhance images. BM3D applies enhanced sparse representation to denoise the images and hence enhance the images to a great extent. However, the low-light condition is not improved much using BM3D. In [2], a contrast enhancement estimation for a digital image forensics method is proposed. However, the authors deal with contrast enhancement for image enhancement more rather than dealing with the real low-light condition. In [3], the extreme low-light image denoising problem is dealt with. The extensive result shows improvement in enhancing images from poor low-light conditions. However, the algorithm lacks speed at inference time and does not deal with the issue of losing details of the images. In [4], a robust retinex model is introduced which introduces an optimization function to improve images from noisy and low-light conditions by providing an augmented Lagrange multiplier-based alternating-direction minimization algorithm without logarithmic transformation. In [5], a low-light image enhancement method, LIME, is proposed, which estimates the illumination of each pixel by computing the maximum value in different channels separately and then finally estimates the illumination map by imposing a structure prior on it. The method exhibits a good result in terms of high-quality exposure enhancement.

In recent years, image enhancement solutions that are deep learning-based have made quite a good impression in research. The deep learning-based methods tend to show more improvements in accuracy than the conventional methods. As deep learning methods impose a robust and faster learning network to predict high-quality images, these methods have gained more popularity in enhancing low-light images. The enhancement method in [6] introduces a residual learning-based method incorporating low-light information to predict high-quality images. The result shows improvement in preserving the finer details while improving the low-light exposure.

In [7], for salient object detection purposes, an image enhancement method for low-light images is proposed. The model that is introduced here embeds the physical lighting model into a deep neural network to incorporate degradation of low-light images. Here, the environment’s light is regarded as a point-wise variate and changes with the local content. In [8], a deep bilateral learning method is proposed to improve the image enhancement in real time. The proposed method learns to make local, global, and content-dependent decisions to approximate the desired image transformation. The algorithm provides a faster transformation suited for real-time image enhancement. In [9], a deep local parametric filter-based image enhancement method is proposed. Here, they propose a novel idea to automatically enhance images using learned spatially local filters of three different types. They design a deep neural network, dubbed Deep Local Parametric Filters, that is intended to regress the parameters of these spatially localized filters being automatically applied to enhance the image. The authors of [10] introduced Image-adaptive 3D Lookup Tables for high-performance image enhancement. They propose to learn 3D lookup tables (LUTS) from annotated data. Moreover, the 3D LUT is image-adaptive, which helps it learn multiple-basis 3D LUTs and a small convolutional neural network (CNN) simultaneously in an end-to-end manner.

While these methods significantly improve visual brightness and perceptual quality in low-light images, they primarily operate in the pixel domain and optimize reconstruction or perceptual metrics. Such approaches are not explicitly designed to preserve gradient-domain structures that are critical for feature-based vision algorithms. In particular, feature detectors such as SIFT rely on extrema detection within the Difference-of-Gaussian (DoG) scale-space representation, which depends on stable gradient structures across multiple scales. Pixel-level enhancement techniques may alter these gradients and consequently affect the reliability of key point detection and matching. In contrast, the proposed IllumiSIFT framework adopts a task-driven strategy that directly learns Gaussian-blurred representations for constructing an enhanced DoG pyramid, thereby preserving scale-space structures required for robust SIFT feature detection in low-light images.

Recent research has also explored advanced frameworks for nighttime and low-light image enhancement using variational optimization and transformer-based architectures. For example, VNDHR [11] introduces a variational framework for nighttime image dehazing using hybrid regularization to improve visibility under complex atmospheric degradation. Transformer-based approaches have also been proposed for low-light enhancement. For instance, CodedBGT [12] utilizes a code bank-guided transformer architecture to capture global illumination relationships and improve enhancement performance. While these methods demonstrate strong capability for perceptual restoration and global illumination correction, they primarily focus on improving visual brightness and pixel-domain image quality. In contrast, the proposed IllumiSIFT framework adopts a task-driven strategy that directly targets the Difference-of-Gaussian (DoG) representation used in SIFT feature detection, emphasizing gradient-domain stability rather than pixel-domain visual reconstruction.

Recent adaptation of generative models like diffusion for low-light image enhancement has also demonstrated potential. In [13], the authors presented a Global Structure-Aware Diffusion Process (GSAD) for low-light image enhancement, which regularizes the diffusion model’s trajectory using non-local structural information to achieve better image quality, noise suppression, and contrast amplification. In another work [14], a Wavelet-based Conditional Diffusion Model (WCDM) for low-light image enhancement was presented that significantly accelerates inference by operating in the wavelet domain. This approach efficiently enhances visual quality and detail while maintaining the generative power of diffusion models.

While effective at denoising and detail restoration, diffusion models often introduce global degradations like color shifts due to the limited local receptive fields. The issue with limited receptive field was addressed in TransDiff [15], which featured a structure-aware extraction stream to recover local details and a lightweight Transformer-based denoising stream to correct global color biases, yielding high-quality results. A recognized limitation, however, is that the generative diffusion process combined with the complex dual-stream architecture inherently results in slower inference speed and a larger model size compared to conventional enhancement methods.

In another study [16], besides improving low-light image enhancement, the authors targeted faster inference on edge devices by training a lightweight U-net based CNN model and performing post-training quantization (downgrading double precision floating weight to INT8). The authors briefly discussed the recent research on the combination of CNN architectures with transformers and why those approaches were not practical as edge vision applications due to their high computational costs.

However, all these methods contribute to improving PSNR rather than preserving the feature points, as most of them have the loss function based on mean square error (MSE). So, from a practical point of view, these enhancement methods generate a more eye-soothing high-exposure image by increasing PSNR, which eventually contributes towards losing key features. The reason is that the loss function based on MSE in the pixel domain only tries to increase the PSNR and makes it visually better. But when we try to detect those images, we have to preserve the important local and global features. Here, the solution would be to design a network that places more emphasis on preserving the features that contribute towards better recognition and detection of robust objects. For example, images that are captured from surveillance cameras have the problem of low-light conditions during nighttime or when there is not enough light in that place. These low-light images have very low-intensity pixel values, which means that they have less information while being identified. While enhancing those images, we also need to be very efficient in preserving the features. Otherwise, the identification will be corrupted. In the Air Force, while detecting any aircraft, the accuracy of detection should be very high. So the quality of the image should be enhanced with high-quality light conditions whilst simultaneously not losing the key features. In short, the enhancement of images whilst preserving the key features/points is of paramount importance. There are several works that improve the features of low-quality images. In [17,18], a low-light enhancement model is proposed to develop recognition of objects. However, we proposed an image enhancement method that aims to preserve the key features by enhancing the low-light dark images in the gradient image domain.

In general, gradient images provide important information derived from pixel images. By definition, gradient images generally refer to a change in the direction of the intensity or color of an image. Numerous works regarding image recognition have been done using the gradient of images. In [19], Harris Detector is used to determine the edges and extract corners of the image and to infer features of the image. In [20], Laplacian of Gaussian is used for blob detection. In [21], SIFT feature detection is used, which discovers local features after computing maxima and minima from the DoG image set. In recognition, key points from an object are extracted to provide a description of the features. So, we should keep in mind that extracted features should be able to be used in case of scale, noise, and illumination changes. Using SIFT, these changes can be handled, which makes SIFT an ideal method for feature extraction. In [22], a visual query compression for preserving local features is introduced. Here, the authors go through a new method of visual key point compression, which uses subspaces for optimization of preserving key point feature matching properties rather than the reconstruction performance. Moreover, SIFT feature preservation plays an important role in image recognition. There are numerous studies on the role of SIFT features in increasing the accuracy of image recognition. In [23,24], the application of SIFT features in image recognition is explained. In [25,26], a gradient image super-resolution method is proposed which improves the SIFT matching points. The proposed method here improves the image resolution in the gradient domain and produces more SIFT key points.

Despite the effectiveness of existing low-light image enhancement methods, a fundamental limitation remains in their reliance on pixel-domain optimization objectives, which prioritize visual appearance rather than feature stability. In practical recognition pipelines, handcrafted local descriptors such as Scale-Invariant Feature Transform (SIFT) rely on gradient extrema derived from the Difference-of-Gaussian (DoG) pyramid, making the preservation of multi-scale gradient information more critical than pixel-level fidelity. Enhancing images solely in the intensity domain does not guarantee that these gradient structures are retained, and in many cases, such enhancement suppresses or distorts local extrema essential for reliable key point detection. Motivated by this observation, this work introduces IllumiSIFT, a task-driven low-light enhancement framework that reframes image enhancement as a gradient-domain learning problem by directly targeting the DoG pyramid used in SIFT-based recognition.

Despite significant advances in low-light image enhancement, most existing methods are optimized in the pixel domain using metrics such as PSNR and SSIM. While these approaches improve visual quality, they do not explicitly preserve gradient-domain structures that are critical for downstream feature detection and matching. In particular, handcrafted feature extractors such as SIFT rely on extrema detection in the Difference-of-Gaussian (DoG) pyramid, yet existing enhancement frameworks do not directly align their learning objectives with this scale-space representation. Therefore, there remains a gap in task-driven low-light enhancement methods that explicitly target gradient-domain stability for feature preservation.

The main contributions of this work are summarized as follows:

- We propose IllumiSIFT, a task-driven low-light enhancement framework that directly learns the Difference-of-Gaussian (DoG) pyramid rather than optimizing conventional pixel-domain reconstruction metrics.
- We introduce a cascaded residual learning architecture with Squeeze-and-Excitation (SE) modules that progressively predicts Gaussian-blurred representations at multiple σ levels, enabling enhanced multi-scale gradient responses aligned with SIFT detection.
- We design a combined objective function incorporating both Gaussian-domain and DoG-domain supervision to preserve scale-space consistency.
- We demonstrate through extensive experiments on the CDVS, Oxford Buildings, and Paris datasets that the proposed approach significantly improves SIFT matching performance under severe low-light conditions.

The proposed framework is not designed as a conventional end-to-end perceptual enhancement system for image enhancement of low-light images. Rather, it is an image enhancement network that generates more matching SIFT points. So, the objective is to enhance the low-light dark images in the gradient domain so that it preserves SIFT features which will eventually contribute to better recognition. Our deep learning network is constructed based on the concept of generating gradient images. The network consists of cascaded image enhancement networks. For each of the networks, we establish a deep learning method inspired by residual learning and Squeeze and Excitation Network [27], but instead of producing the enhanced high-exposure images of original low-light inputs, we produce the Gaussian-blurred images in the stages of the cascaded network with different standard deviations to finally compute the Difference of Gaussian. In SIFT, the Difference of Gaussian (DoG) images are produced from the input image with different scales and different standard deviations. In our method, the network produces the Gaussian-blurred images in its different stages of the cascaded network with similar standard deviations and computes the DoG images, and then integrates with SIFT method to find the key points which are used for matching. Usually, DoG images from dark images have less information. That is why it is difficult to determine the maximum and minimum points that indicate corner or edge points as well as key points. In brief, our proposed method aims at generating enhanced gradient images that increase the matching SIFT feature points, hence producing SIFT repeatability.

The remainder of this paper is organized as follows. Section 2 describes the proposed IllumiSIFT framework and the cascaded architecture used to generate Gaussian-blurred representations for constructing the enhanced DoG pyramid. Section 3 presents the experimental setup and evaluation methodology. Section 4 reports and analyzes the experimental results followed by Section 5 presenting a discussion. Finally, Section 6 concludes the paper and discusses potential directions for future research.

## 2. Proposed Method

We propose a novel method that consists of a deep learning pipeline for dark image enhancement. In our work, our objective is to produce more SIFT matching points that will bear the evidence of preserving features. So, instead of enhancing low-light dark images with a pixel-level recovery loss, we generate enhanced Gaussian-blurred images with different standard deviations, i.e., the DoG pyramid as our learning target. The purpose is to produce enhanced gradient images from the Gaussian-blurred image to finally integrate with SIFT to preserve SIFT matching points. Our proposed image enhancement infrastructure is built upon the basis of producing enhanced gradient images. Basically, gradient images are produced from the original image being convolved with a filter. In a gradient image, e.g., DoG image in our case, in a certain direction, each pixel determines the change in intensity of that same point in the original image. Our image gradient method is synced with the SIFT method. In the SIFT method, different Gaussian-blurred images are first produced with different standard deviations from the input images in different scales. Then the Difference of Gaussian (DoG) is computed for different scales which are called octaves. With a view to finding key feature points, maxima and minima are generated to find key points from the DoG images. Later, the edges and low-contrast points are eliminated, considering them as bad points. With rotation and scale invariance being considered, the key points are detected. *P*(*x*,*y*) is the original image; *G*(*x*,*y*,*σ*) is the Gaussian Kernel. Equations (1) and (2) show the formulation of Gaussian-blurred images.

(1) $$G\left(x,y,\sigma \right)=\frac{1}{2\pi \sigma^{2}}e^{\frac{-(x^{2}+y^{2})}{2\sigma^{2}}}$$

(2) B(x,y,σ)=G(x,y,σ)∗P(x,y)

where *B*(*x*,*y*,σ) is the Gaussian-blurred image with specific σ, which is the standard deviation; *x* is the distance from the origin in the horizontal axis; *y* is the distance from the origin in the vertical axis. So, the Difference of Gaussian will be as follows in Equations (3) and (4):

(3) $$D\left(x,y,\sigma_{1},\sigma_{2}\right)=\left(G_{1}\left(x,y,\sigma_{1}\right)-G_{2}\left(x,y,\sigma_{2}\right)\right)*P\left(x,y\right))$$

(4) $$D\left(x,y,\sigma_{1},\sigma_{2}\right)=B_{1}\left(x,y,\sigma_{1}\right)-B_{2}\left(x,y,\sigma_{2}\right)$$

where *D*(*x*,*y*,$\sigma_{1}$,$\sigma_{2}$) is the Difference of Gaussian, $\sigma_{1}$ is the standard deviation of the first blurred image and $\sigma_{2}$ is the standard deviation of the second blurred image. $G_{1}$ and $G_{2}$ are Gaussian filters. $B_{1}$ and $B_{2}$ are Gaussian-blurred images. The loss function *M* is the L1 loss between the DoG of the enhanced blurred generated image and the DoG from convolution with the original image, which can be shown in (5):

(5) $$M\left(\hat{D},D_{original}\right)=\sum_{i=1}^{H}\sum_{j=1}^{W}\left({\hat{D}}^{ij}-D_{original}^{ij}\;\right)$$

where $\hat{D}$ is the predicted DoG image which is enhanced and $D_{original}$ is the DoG image computed from the original one convolved with a Gaussian filter. H and W are the numbers of pixels in x and y directions.

**Figure 1:**
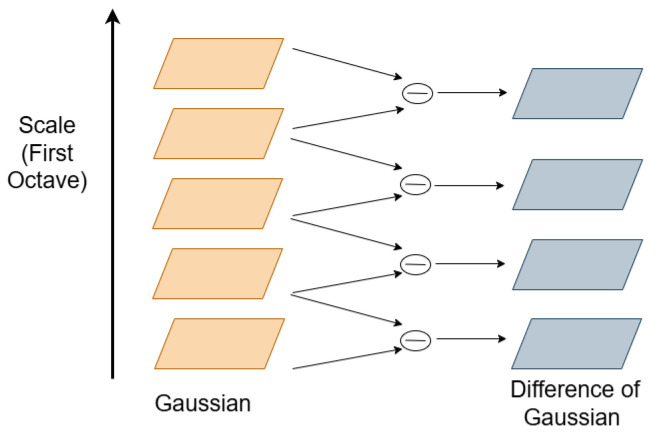
Differenceof Gaussian.

In SIFT key point detection, the DoG images at different scales are generated to produce the DoG Pyramid, and hence using this DOG hierarchy, local maxima and minima are computed and sorted to detect the key points. Figure 1 shows the DoG pyramid at different scales of Gaussian-blurred images.

### 2.1. Network Architecture

Our proposed method is very precise and simple. From the dark input images, the deep learning-based gradient image enhancement stage creates enhanced Gaussian-blurred images, which in turn produces the DoG images. The SIFT integration stage integrates the DoG images for enhancing SIFT matching points. As SIFT needs a DoG pyramid at different scales, our goal is to compute this DoG pyramid with DoG images. We introduce a cascaded network to predict the Gaussian-blurred images at different stages of the cascaded output instead of learning all the Gaussian-blurred images separately.

**Figure 2:**
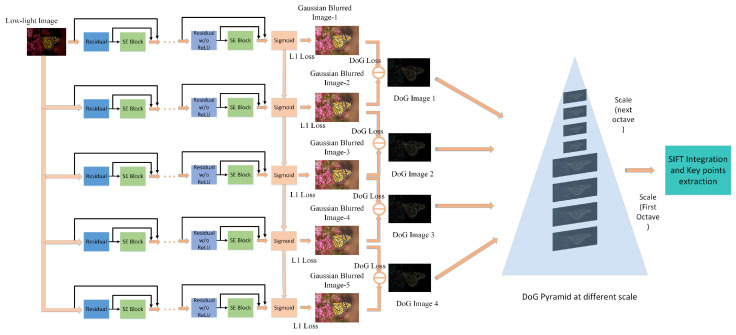
Cascadedsequential architecture for progressive multi-scale Gaussian prediction. Each stage refines scale-specific representations with decreasing σ values.

Figure 2 shows the full network architecture of our proposed method. The purpose of our network is to create five Gaussian-blurred images and then compute DoG images from them. For image enhancement here, we use the series of residual blocks to predict the first Gaussian-blurred image. The residual blocks are not used for direct perceptual enhancement. Instead, they facilitate stable learning of scale-specific Gaussian representations by modeling residual mappings between the dark input and its corresponding blurred representation. This residual formulation enables efficient gradient propagation and preserves structural information necessary for accurate DoG generation. In each stage, the input dark image is fed to the layers. The weights and coefficients from the sigmoid layer before predicting the first Gaussian-blurred images are reused and concatenated to the sigmoid layer of the new cascaded layer. The input dark image is again fed to the second cascaded layer, which then consists of a series of residual blocks to predict the next Gaussian-blurred image. We apply similar cascaded architecture to predict the other three Gaussian-blurred images. Thus, in each cascaded stage, the weights from the previous layer are fed at their own sigmoid layer. It is important to clarify that the architecture shown in Figure 2 operates in a cascaded sequential manner rather than parallel processing. Each stage receives the original dark input image along with feature representations propagated from the previous stage. The stages are executed progressively, where the output of one stage influences the parameter reuse and refinement of the next stage. Therefore, the network does not consist of independent parallel branches but instead forms a progressive multi-scale cascade. Although the same dark input image is reintroduced at each stage, the outputs of different channels are not identical. Each cascaded stage predicts a Gaussian-blurred representation corresponding to a different standard deviation σ. The network parameters and feature representations evolve across stages through parameter reuse and feature propagation, which results in distinct Gaussian predictions at each scale. Consequently, the resulting DoG images represent different levels of scale-space information rather than identical outputs from parallel processing.

**Figure 3:**
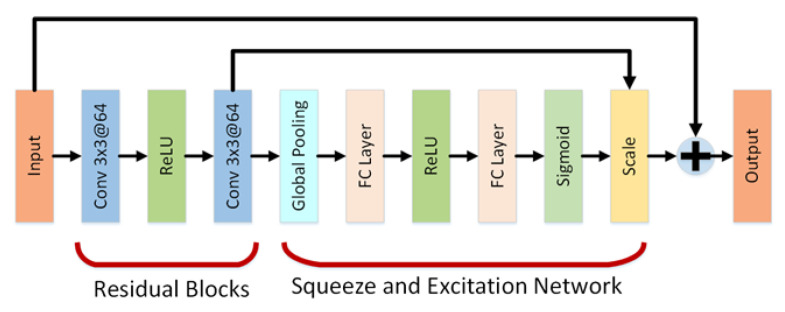
Residual Blocks with Squeeze and Excitation Network.

Our network is a cascaded architecture with a continuation of residual blocks to learn the Gaussian images with different sigmas at different stages. The design of the number of residual blocks and choice of sigma for the prediction of Gaussian-blurred images are discussed in Section 2.3. However, we also add a Squeeze and Excitation network with each residual block. The residual blocks’ architecture is shown in Figure 3. Residual blocks have a convolutional layer followed by a rectified linear unit (ReLU) and again a convolutional layer. Each convolutional layer has a filter kernel size of 3 × 3 with 64 features. In the Squeeze and Excitation network, the output from the residual block is followed by a global pooling layer, a fully connected layer, ReLU, a fully connected layer again and a sigmoid function followed by scaling. The input to the residual block is added to the output of the Squeeze and Excitation network for residual learning. The Squeeze and Excitation network is added to develop the feature responses of the channels by modelling the relationships between channels [27] working as a boosting factor in our method. However, the final convolution within each residual block does not employ a ReLU activation. This design choice preserves the signed nature of the predicted Gaussian and DoG representations, since both positive and negative gradient responses are essential for accurate extrema detection in the SIFT pipeline. Introducing a non-linear activation such as ReLU at the final stage could truncate negative values and distort the gradient-domain information.

**Figure 4:**
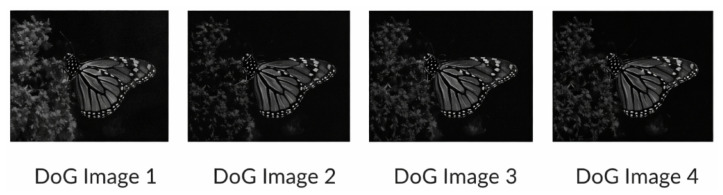
The four DoG images.

Figure 4 illustrates visual examples of the generated DoG images at different scale levels. As the scale decreases, finer gradient structures become more prominent, demonstrating the progressive refinement capability of the cascaded network. As we generate the DoG images computed from the generated enhanced high-light-exposure blurred images from the network, we merge them into the SIFT network. In SIFT, the DoG images are computed from the Gaussian-blurred images with different σ values in different scaling octaves. Our network produces the blurred images. So, in our case, we directly load our DoG image pyramid into the SIFT network. So, the SIFT network will find key points from our produced DoG images. The purpose of integrating with SIFT is that SIFT itself computes DoGs on different scales to determine the maxima and minima in DoG images for identifying key points. Since the proposed network generates enhanced multi-scale DoG representations with stronger gradient responses, the subsequent SIFT extrema detection process becomes more stable. This allows key points to be detected more reliably without suppressing the structural features necessary for matching. Thus the integration of our DoG images with SIFT actually aims at preserving key features. Moreover, it is to be noted that the predicted Gaussian-blurred images are used to construct the enhanced Difference-of-Gaussian (DoG) pyramid. This DoG pyramid replaces the internally computed DoG in the standard SIFT pipeline. All subsequent SIFT operations, including extrema detection, key point localization, orientation assignment, and descriptor computation, are performed without modification. Therefore, our method enhances only the gradient-domain representation while preserving the original SIFT feature extraction procedure.

### 2.2. Loss Function

We combine two types of losses in our method: L1 loss and DoG loss. L1 loss is calculated between the predicted Gaussian-blurred images and the original image convolved with the Gaussian filter at each cascaded output stage. The DoG loss is calculated between the DoG images from two consecutive cascaded output stages and the original DoG image after the convolution with two different Gaussian filters of the corresponding standard deviation.

For illustrative purposes, we present a generalized gradient formulation to show how the loss depends on the predicted DoG representations. The detailed gradients are computed automatically via backpropagation during training.

(6) $$\frac{\partial M}{\partial \hat{D}}=\frac{\partial (\sum_{i=1}^{H}\sum_{j=1}^{W}\left({\hat{D}}^{ij}-D_{original}^{ij}\;\right))}{\partial \hat{D}}$$

(7) $$\frac{\partial M}{\partial \hat{D}}=\sum_{i=1}^{H}\sum_{j=1}^{W}\left(1-\left(\frac{1}{2\pi {\sigma_{1}}^{2}}\frac{\partial A}{\partial \hat{D}}-\frac{1}{2\pi {\sigma_{2}}^{2}}\frac{\partial B}{\partial \hat{D}}\right)\right)$$

(8) $$A=e^{\frac{-(x_{i}^{2}+y_{j}^{2})}{2{\sigma_{1}}^{2}}}*P\left(x_{i},y_{j}\right),B=e^{\frac{-(x_{i}^{2}+y_{j}^{2})}{2{\sigma_{2}}^{2}}}*P\left(x_{i},y_{j}\right)$$

Here, Equation (7) is derived from Equation (6) after differentiating it with respect to $\hat{D}$. In Equation (8), $x_{i}$ is the distance from the origin in the horizontal axis, and $y_{j}$ is the distance from the origin in the vertical axis. The loss function can be simplified if we use the L1 loss between Gaussian-blurred images as our loss function and then we compute the DoG images from the Gaussian-blurred image. Equation (9) is the simplified L1 loss function:

(9) $$M_{1}\left(\hat{L},L_{original}\right)=\sum_{i=1}^{H}\sum_{j=1}^{W}\left({\hat{L}}^{ij}-L_{original}^{ij}\;\right)$$

where $\hat{L}$ is the predicted blurred image which is enhanced and $L_{original}$ is the Gaussian-blurred image of the original image with the same standard deviation. In our proposed method, this is presented along with the L1 loss.

The second loss function DoG can be described from (5). For clarity in the final objective formulation, we explicitly denote the DoG loss component as M2, which follows the same L1 formulation introduced in Equation (5).

(10) $$M_{2}\left(\hat{D},D_{original}\right)=\sum_{i=1}^{H}\sum_{j=1}^{W}\left({\hat{D}}^{ij}-D_{original}^{ij}\;\right)$$

Finally, if we combine Equations (9) and (10), our final loss function becomes

(11) $$Loss=M_{1}+M_{2}$$

Initially, while starting the learning process, the network only learns through L1 loss as we do not have any DoG image prediction to compute the DoG loss. As the network starts learning Gaussian-blurred images, hence computing the DoG images, the network combines both L1 loss and DoG loss.

### 2.3. Training Strategy

From our network architecture, it is evident that the first predicted Gaussian-blurred image needs the lowest number of residual blocks for prediction. It basically indicates that it is better to predict the Gaussian-blurred image with lesser information in the first stage and then, with the increase in the number of residual blocks, we can predict Gaussian-blurred images with more information. We know that the Gaussian-blurred images should be at different values of σ. So, we predict the Gaussian-blurred images with larger sigma first. As we increase the number of residual blocks, we learn Gaussian-blurred images with smaller σ.

In order to choose the number of residual blocks, we analyzed the radially averaged power spectrum of the ground truth image and the Gaussian-blurred images with different sigma. To quantitatively analyze the scale-dependent information variation across Gaussian-blurred images, we examine the radially averaged power spectrum. Since Gaussian smoothing progressively attenuates high-frequency components as σ increases, analyzing the frequency-domain energy distribution provides an objective measure of information loss at different scales. Radial averaging ensures isotropic aggregation of frequency magnitudes, avoiding directional bias in the analysis. This frequency-domain characterization guided the architectural design choice of progressively increasing network depth for predicting Gaussian representations with smaller σ values.

(12) $$P\left(r\right)=\frac{1}{|\Omega_{r}|}\sum_{\left(u,v\right)\in \Omega_{r}}{\left|F(u,v)\right|}^{2}$$

where F(u,v) denotes the Fourier transform of the image, $\Omega_{r}=\left\{\left(u,v\right):\sqrt{u^{2}+v^{2}}=r\right\}$ represents the set of frequency coordinates at radial distance *r*, and $|\Omega_{r}|$ denotes the number of frequency samples in that radial band.

**Figure 5:**
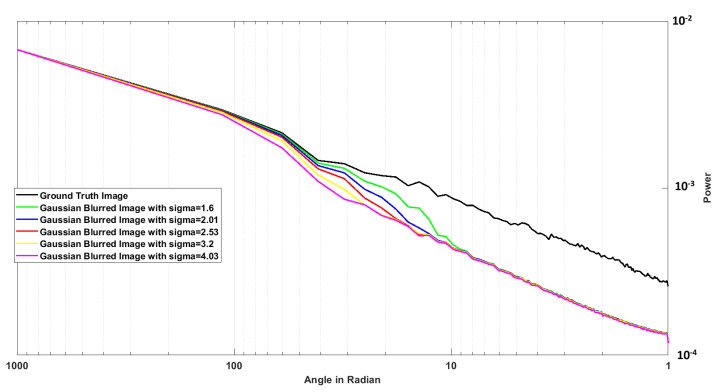
Radially Averaged Power Spectrum of the ground truth image and the Gaussian-blurred images.

Figure 5 shows the plot of the radially averaged power spectrum. From the plot, we can see that as we increase the value of sigma, the power falls. The reason for this is that with higher values of sigma of Gaussian-blurred images, the information tends to reduce. So, images with larger sigma have less information. So, in our design, we predict the Gaussian-blurred image with larger sigma first with fewer residual blocks. As we decrease the value of sigma, we add more residual blocks along with the weights and coefficients from the previous layer to predict images with more information.

In the first stage, we use six residual blocks to predict the first Gaussian-blurred image in the cascaded network. For the following stages, we add two more residual blocks in each cascaded stage to predict the Gaussian-blurred image. As we concatenate the weights and coefficients of each layer to the next one, the number of residual blocks to predict the Gaussian-blurred images could be viewed as 6, 8, 10, 12, and 14 collectively. However, the choice of sigma is made in accordance with the original design of SIFT. We choose the σ values of 4.03, 3.2, 2.53, 2.01, and 1.6 in accordance with the design of SIFT which is shown Table 1. The naming of the σ is done with the smallest sigma as $\sigma_{1}$ and so on. Table 2 illustrates the full process.

## 3. Experimental Setup and Dataset

The proposed model is trained using the DIV2K dataset, which contains 800 high-resolution images widely used for image restoration and enhancement research. For evaluation, we use three benchmark datasets commonly employed in feature matching and image retrieval studies: the MPEG Compact Descriptors for Visual Search (CDVS) dataset, the Oxford Buildings dataset, and the Paris dataset. These datasets provide diverse image pairs suitable for evaluating SIFT key point detection and matching performance under varying illumination conditions. While DIV2K provides sufficient training data for learning the enhancement model, the CDVS, Oxford, and Paris datasets are widely used benchmarks for evaluating feature repeatability and image matching performance. The datasets used in this work are publicly available. The DIV2K dataset can be accessed at https://data.vision.ee.ethz.ch/cvl/DIV2K/ (accessed on 18 March 2026). The MPEG CDVS dataset is available at https://mpeg.chiariglione.org/standards/mpeg-7/compact-descriptors-visual-search/n14960-working-draft-2-cdvs-conformance-testing.html (accessed on 18 March 2026). The Oxford Buildings dataset is available at https://www.robots.ox.ac.uk/~vgg/data/oxbuildings/ (accessed on 18 March 2026), and the Paris Buildings dataset is available at https://www.robots.ox.ac.uk/~vgg/data/parisbuildings/ (accessed on 18 March 2026).

**Figure 6:**
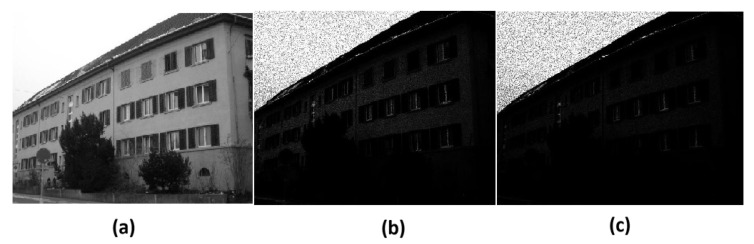
Comparison of low-light image enhancement using gamma correction. (**a**) Original dark image. (**b**) Gamma = 5 corrected image. (**c**) Gamma = 7 corrected image. Increasing the gamma value improves brightness but may distort gradient structures and suppress fine details, which can negatively affect SIFT key point detection.

A. Training Dataset:

For training, we used the CVPR DIV 2K dataset [28] with 800 training images. We only used the grayscale images for our experiment. We add a gamma correction to the input images to make them dark. We applied gamma = 5 and gamma = 7 to the input images and then mixed them all together to make the dataset robust. We then added poison noise to the images to make them noisy. The input dark images are then cropped to 32 × 32 patch size. Figure 6 shows the original image and gamma-corrected images in grayscale.

The training process is conducted on a computer equipped with an Intel core I-7 at 3.2 GHz with 32 GB memory with GPU. The coding platform we used here is Python 3.10 with the PyTorch [29] deep learning tool. We implemented the architecture and performed processing in PyTorch.

**Figure 7:**
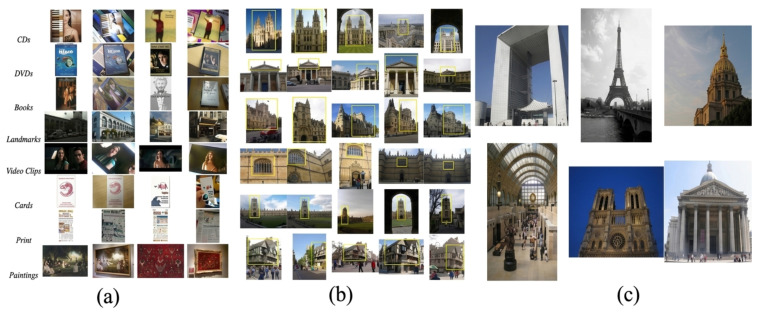
Experimental Datasets. (**a**) CDVS Dataset, (**b**) Oxford Building Dataset, (**c**) Paris dataset.

B. Testing Dataset:

For testing, we used the MPEG Compact Descriptors for Visual Search (CDVS) dataset [30], Oxford building dataset [31] and Paris dataset [32]. CDVS is a comprehensive collection of images of various objects, which consists of 186k labeled images of CDs and book covers, paintings, video frames, buildings and common objects, as shown in Figure 7a. The Oxford building dataset has 5062 images with 55 queries, as shown in Figure 7b, and the Paris dataset has 6412 images with 12 queries, as shown in Figure 7c. We experimented after choosing 200 matching image pairs from each dataset. Similar to the training dataset, we applied gamma correction (5 and 7) to the dataset first. We used our trained networks for generating the DoG images and then integrated them into SIFT. For performance evaluation, we show the number of SIFT matching points.

## 4. Results

For the evaluation of the performance, we basically compare our result with LIME, HDRnet, Retinex, Deep LPF, and 3D-LUT. We tested the method against CDVS, Oxford, and Paris datasets with 200 matching image pairs from each one. We compared the PSNR of our predicted DoG images with the DoG images produced from LIME, HDRnet, Retinex, Deep LPF and 3D-LUT for gamma = 5 and gamma = 7 corrected noisy images.

Table 3 shows the result for PSNR in dB for four DoG images generated blurred at $\sigma_{k}$ and $\sigma_{k+1}$ (*k* = 1, 2, 3, 4, 5) using our proposed methods for the CDVS full dataset. DoG images generated from LIME images, HDRnet images, Retinex images, Deep LPF images and 3D-LUT images were all convolved with Gaussian filters for the CDVS full dataset. It is seen that DoG images from our proposed method have acquired around 0.8–1 dB gain over the DoG images generated from LIME convolved with Gaussian filters, 1.2–1.4 dB gain over the DoG images generated from HDRnet, 1.8–2 dB gain over the DoG images generated from Retinex, 0.6–0.8 dB gain over the DoG images generated from Deep LPF and 0.9–1.1 dB gain over the DoG images generated from 3D-LUT method.

Table 4 shows the result for PSNR in dB for four DoG images generated blurred at $\sigma_{k}$ and $\sigma_{k+1}$ (*k* = 1, 2, 3, 4, 5) using our proposed methods for the Oxford dataset. DoG images generated from LIME images, HDRnet images, Retinex images, Deep LPF, and 3D-LUT images were all convolved with Gaussian filters for the CDVS full dataset. It is crystal clear that DoG images from our proposed method have acquired around 0.8–1.2 dB gain over the DoG images generated from LIME convolved with the Gaussian filter, 1–1.5 dB gain over the DoG images generated from HDRnet, 1.8–2.2 dB gain over the DoG images generated from Retinex, 1–1.1 dB gain over the DoG images generated from Deep LPF and 1–1.1 dB gain over the DoG images generated from 3D-LUT method.

Table 5 shows the result for PSNR in dB for four DoG images generated blurred at $\sigma_{k}$ and $\sigma_{k+1}$ (*k* = 1, 2, 3, 4, 5) using our proposed methods for the Paris dataset. DoG images generated from LIME images, HDRnet images, Retinex images, Deep LPF images and 3D-LUT images were all convolved with Gaussian filters for the CDVS full dataset. It is crystal clear that DoG images from our proposed method have acquired around 0.8–1.2 dB gain over the DoG images generated from LIME convolved with the Gaussian filter, 1.1–1.5 dB gain over the DoG images generated from HDRnet, 1.7–2.1 dB gain over the DoG images generated from Retinex, 0.7–0.8 dB gain over the DoG images generated from Deep LPF and 1.1–1.2 dB gain over the DoG images generated from 3D-LUT method.

**Figure 8:**
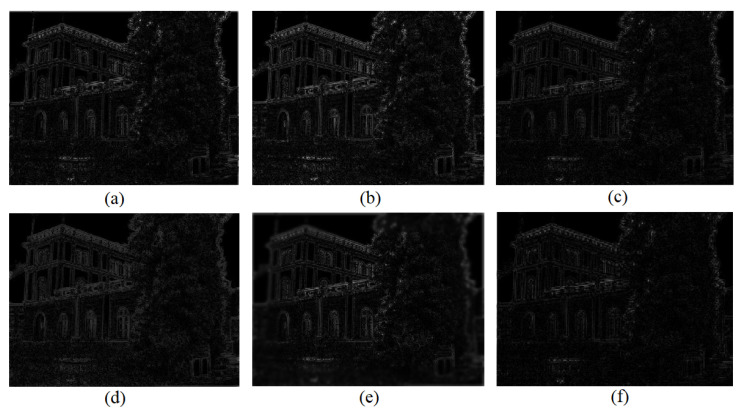
DoG PSNR comparison using different models. (**a**) DoG image generated from original image. (**b**) DoG image using proposed method (52.35 dB PSNR). (**c**) DoG image using Deep LPF (49.18 dB PSNR). (**d**) DoG image using LIME (47.27 dB PSNR). (**e**) DoG image using 3D-LUT (47.11 dB PSNR). (**f**) DoG image using Retinex model (45.75 dB PSNR).

Figure 8 shows the images of the DoG at sigma 1.6 and 2.1 produced using different image enhancement methods. From the images, it is clear that our proposed method has produced the best DOG image in terms of PSNR.

We then integrate the DoG images into the SIFT. After the integration, we compute SIFT matching points and compare our results with dark images, LIME, HDRnet, Retinex, Deep LPF and 3D-LUT.

We first evaluate performance on the CDVS dataset to analyze large-scale retrieval robustness under low-light degradation. Table 6 shows the average SIFT matching points of 200 image pairs for the CDVS full dataset for gamma = 5 and gamma = 7 corrected images separately. For gamma = 5, our proposed method has a 19-point gain over dark images, a 6-point gain over LIME, a 10-point gain over dark HDRnet, a 13-point gain over Retinex, a 7-point gain over Deep LPF, and an 8-point gain over 3D-LUT. For gamma = 7, our proposed method has a 22-point gain over dark images, a 7-point gain over LIME, a 9-point gain over dark HDRnet, an 11-point gain over Retinex, a 7-point gain over Deep LPF, and a 7-point gain over 3D-LUT. So, it is obvious that our proposed method is able to detect more SIFT key matching points than the other methods.

We further assess landmark recognition performance on the Oxford Buildings dataset. Table 7 shows the average SIFT matching points of 200 image pairs for the Oxford dataset for gamma = 5 and gamma = 7 corrected images separately. For gamma = 5, our proposed method has an 18-point gain over dark images, a 6-point gain over LIME, a 10-point gain over dark HDRnet, a 12-point gain over Retinex, an 8-point gain over Deep LPF, and an 8-point gain over 3D-LUT. For gamma = 7, our proposed method has a 21-point gain over dark images, a 7-point gain over LIME, a 10-point gain over dark HDRnet, a 13-point gain over Retinex, an 8-point gain over Deep LPF, and a 10-point gain over 3D-LUT. So, it is obvious that our proposed method is able to detect more SIFT key matching points than the other methods.

Then, we validate matching consistency on the Paris dataset. Table 8 shows the average SIFT matching points of 200 image pairs for the Paris dataset for gamma = 5 and gamma = 7 corrected images separately. For gamma = 5, our proposed method has a 15-point gain over dark images, a 5-point gain over LIME, an 8-point gain over dark HDRnet, an 11-point gain over Retinex, a 7-point gain over Deep LPF, and an 8-point gain over 3D-LUT. For gamma = 7, our proposed method has a 20-point gain over dark images, a 5-point gain over LIME, an 10-point gain over dark HDRnet, a 12-point gain over Retinex, a 7-point gain over Deep LPF, and a 7-point gain over 3D-LUT. So, it is obvious that our proposed method is able to detect more SIFT key matching points than the other methods.

**Figure 9:**
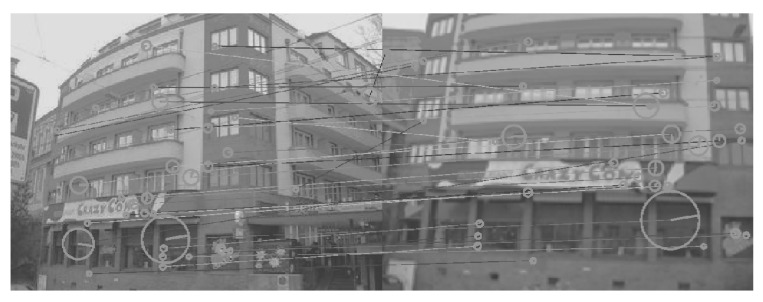
SIFT Comparison using the Retinex Model (40 matching points).

**Figure 10:**
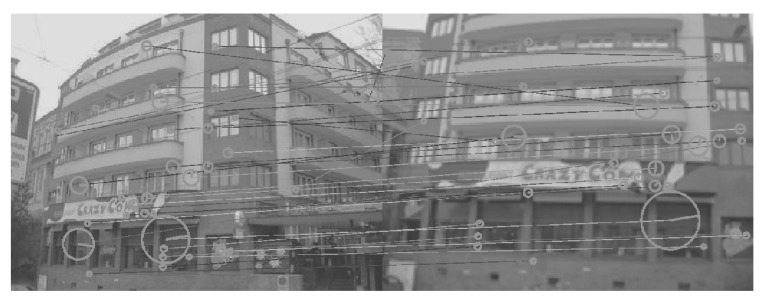
SIFT Comparison using HDRnet (42 matching points).

**Figure 11:**
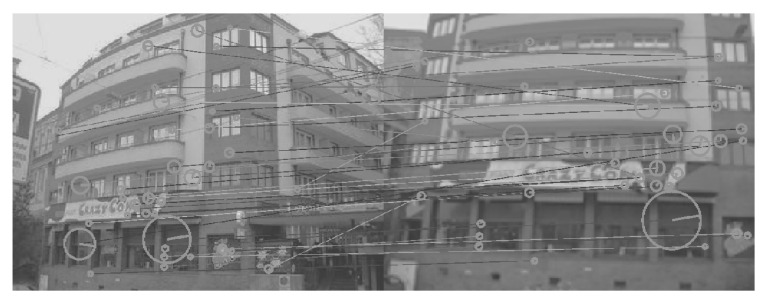
SIFT Comparison using 3D LUT (45 matching points).

**Figure 12:**
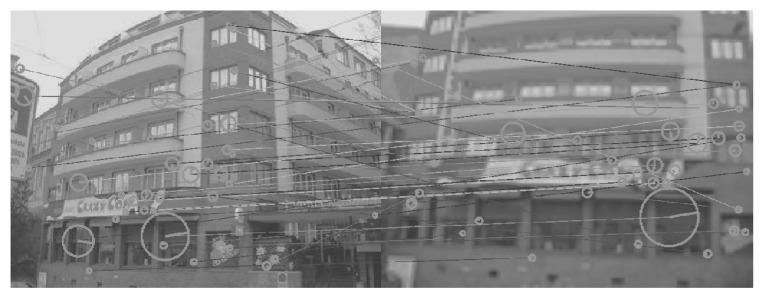
SIFT Comparison using DEEP LPF (46 matching points).

**Figure 13:**
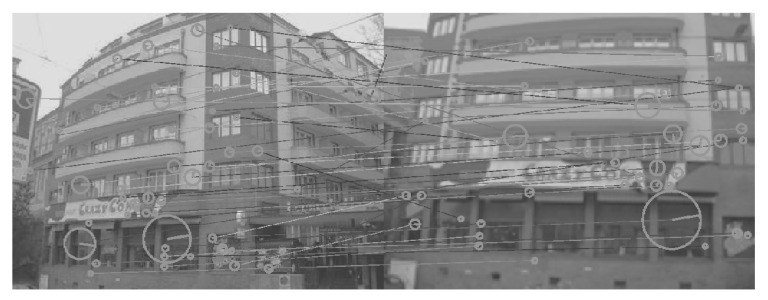
SIFT Comparison using LIME (48 matching points).

**Figure 14:**
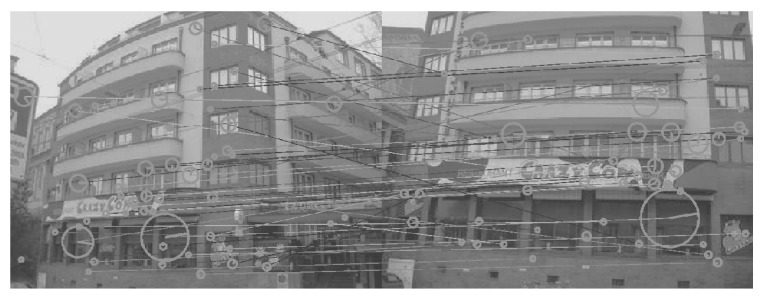
SIFT Comparison using proposed method (55 matching points).

Figure 8, Figure 9, Figure 10, Figure 11, Figure 12, Figure 13 and Figure 14 show the comparative visual results of SIFT matching key points from a building image pair from the CDVS dataset. Our proposed method has the best results with 55 matching points.

### Ablation Analysis

To analyze the contribution of different loss components, we examine the roles of the two loss functions used in the proposed framework. The first loss component (M1) encourages the network to generate Gaussian-blurred images consistent with the target scale-space representation, while the second loss component (M2) enforces consistency between the generated DoG responses and those obtained from the original image.

Using both loss terms jointly allows the network to preserve important gradient structures across multiple scales. Empirically, we observed that combining the two losses stabilizes the training process and improves the quality of the generated DoG representations, which ultimately contributes to better SIFT keypoint detection and matching performance.

## 5. Discussion

The proposed IllumiSIFT framework focuses on preserving gradient-domain structures that are critical for SIFT keypoint detection rather than improving perceptual image quality. Experimental results demonstrate that learning Gaussian-blurred representations enables more stable Difference-of-Gaussian (DoG) responses, which in turn improves keypoint repeatability and matching performance under low-light conditions. Unlike conventional enhancement approaches that optimize pixel-domain metrics such as PSNR or SSIM, the proposed method directly targets the scale-space representation used in SIFT. This task-driven design allows the network to generate enhanced DoG images that preserve extrema structures across multiple scales. As a result, the proposed framework produces a larger number of stable matching keypoint compared to conventional enhancement techniques.

### Limitations

Although the proposed approach improves feature detection stability in low-light conditions, several limitations remain. First, the framework is designed for feature-preserving enhancement rather than perceptual image restoration, and therefore it may not always produce visually pleasing enhanced images. Second, the current model is evaluated primarily on datasets commonly used in feature matching and image retrieval tasks. Future work could extend the evaluation to additional real-world low-light datasets and investigate the generalization of the proposed framework under more diverse degradation conditions.

## 6. Conclusions

Low-light and low-exposure imaging remains a critical bottleneck for reliable visual recognition systems, particularly in surveillance, navigation, and large-scale image retrieval applications where feature stability is more important than visual aesthetics. While recent deep learning-based enhancement methods have demonstrated strong performance in pixel-domain metrics such as PSNR and SSIM, these improvements do not necessarily translate into enhanced downstream machine vision performance. In many cases, aggressive pixel recovery leads to the suppression of local gradients and structural cues that are essential for robust keypoint detection and matching.

In this work, we presented IllumiSIFT, a task-driven gradient-domain enhancement framework that directly targets the preservation and amplification of SIFT-relevant features in low-light images. Unlike conventional enhancement pipelines that operate purely in the image intensity domain, our approach learns to reconstruct the Difference-of-Gaussian (DoG) pyramid directly from dark inputs using a cascaded residual learning architecture. By predicting Gaussian-blurred representations at progressively finer scales and reusing learned features across cascaded stages, the proposed method effectively enhances gradient responses that are critical for stable extrema detection in SIFT.

Extensive experiments conducted on three widely used benchmark datasets, CDVS, Oxford Buildings, and Paris, demonstrate that the proposed framework consistently outperforms state-of-the-art enhancement methods, including LIME, HDRNet, Retinex, Deep LPF, and 3D-LUT. Quantitative evaluations show notable gains in DoG-domain PSNR across multiple scales, confirming that the learned gradient representations are closer to those derived from well-exposed images. More importantly, downstream evaluation using SIFT reveals a substantial increase in the number of reliable matching keypoint across all datasets and illumination levels. These results validate the central hypothesis of this work: optimizing enhancement in the gradient domain yields superior machine vision performance compared to pixel-level recovery alone.

Another important advantage of the proposed approach is its architectural efficiency. By employing a cascaded design with progressive complexity and shared parameters, the network avoids the computational overhead associated with learning multiple independent scale representations. This makes the framework well-suited for integration into practical vision pipelines where both performance and efficiency are critical.

While this work focuses on SIFT as a representative handcrafted feature extractor, the underlying concept of task-aligned gradient learning is general and can be extended to other local descriptors and hybrid pipelines. Future work will explore integrating the proposed framework with learning-based local features, evaluating the method on additional real-world low-light datasets (e.g., LOL and ExDark), extending the model to color and multi-spectral imagery, and optimizing the architecture for real-time and edge-device deployment. Additionally, incorporating explicit downstream task losses, such as image retrieval accuracy or pose estimation error, presents a promising direction for further improving task-driven enhancement.

Overall, IllumiSIFT demonstrates that reframing low-light enhancement as a feature-preservation problem rather than a visual reconstruction problem leads to meaningful gains in practical recognition tasks, offering a new perspective for designing enhancement models in machine vision-centric applications.

## Figures and Tables

**Table 1:** Choice of σ.

$\sigma_{1}$	$\sigma_{2}$	$\sigma_{3}$	$\sigma_{4}$	$\sigma_{5}$
1.6	2.01	2.53	3.2	4.03

**Table 2:** Layer configuration of each cascaded stage.

Stage	σ	# Blocks	Output
1	$\sigma_{5}$	6	$G_{1}$
2	$\sigma_{4}$	8	$G_{2}$
3	$\sigma_{3}$	10	$G_{3}$
4	$\sigma_{2}$	12	$G_{4}$
5	$\sigma_{1}$	14	$G_{5}$

**Table 3:** PSNR (in dB) comparison of DoG images for CDVS full dataset.

DoG ($\sigma_{k}$,$\sigma_{k+1}$)	LIME	HDRnet	Retinex	Deep LPF	3D-LUT	Proposed Method
$D_{1}$ ($\sigma_{1}$,$\sigma_{2}$)	24.19	23.86	23.15	24.41	24.15	25.24
$D_{1}$ ($\sigma_{2}$,$\sigma_{3}$)	25.39	25.02	24.27	25.62	25.31	26.43
$D_{2}$ ($\sigma_{3}$,$\sigma_{4}$)	26.55	26.14	25.41	26.8	26.65	27.48
$D_{2}$ ($\sigma_{4}$,$\sigma_{5}$)	28.13	27.77	27.12	28.29	28.02	28.91

**Table 4:** PSNR (in dB) comparison of DoG images for Oxford Dataset.

DoG ($\sigma_{k}$,$\sigma_{k+1}$)	LIME	HDRnet	Retinex	Deep LPF	3D-LUT	Proposed Method
$D_{1}$ ($\sigma_{1}$,$\sigma_{2}$)	25.15	24.55	24.24	25.21	24.94	26.03
$D_{1}$ ($\sigma_{2}$,$\sigma_{3}$)	25.95	25.02	25.32	26.36	25.82	27.25
$D_{2}$ ($\sigma_{3}$,$\sigma_{4}$)	26.98	26.24	26.06	27.31	26.95	28.42
$D_{2}$ ($\sigma_{4}$,$\sigma_{5}$)	28.44	27.62	27.49	28.95	28.61	29.69

**Table 5:** PSNR (in dB) comparison of DoG images for Paris Dataset.

DoG ($\sigma_{k}$,$\sigma_{k+1}$)	LIME	HDRnet	Retinex	Deep LPF	3D-LUT	Proposed Method
$D_{1}$ ($\sigma_{1}$,$\sigma_{2}$)	24.97	24.21	24.1	25.01	24.69	25.75
$D_{1}$ ($\sigma_{2}$,$\sigma_{3}$)	25.78	24.77	25.11	25.91	25.55	27.04
$D_{2}$ ($\sigma_{3}$,$\sigma_{4}$)	26.84	26.11	25.92	27.14	26.68	28.18
$D_{2}$ ($\sigma_{4}$,$\sigma_{5}$)	28.31	27.42	27.36	28.82	28.33	29.51

**Table 6:** Average number of SIFT matching points computed over 200 image pairs from the CDVS dataset under simulated low-light degradation. The CDVS dataset evaluates large-scale visual retrieval robustness in challenging illumination conditions.

Gamma Value	Dark Images	LIME	HDRnet	Retinex	Deep LPF	3D-LUT	Proposed Method	Original Images
5	33	46	42	39	45	44	52	106
7	26	41	39	37	41	41	48	106

**Table 7:** Average number of SIFT matching points on the Oxford Buildings dataset, evaluating landmark recognition performance under low-light conditions.

Gamma Value	Dark Images	LIME	HDRnet	Retinex	Deep LPF	3D-LUT	Proposed Method	Original Images
5	30	42	38	36	40	40	48	94
7	24	38	35	32	37	35	45	94

**Table 8:** Average number of SIFT matching points on the Paris dataset, assessing repeatability and matching consistency across diverse urban scenes under illumination degradation.

Gamma Value	Dark Images	LIME	HDRnet	Retinex	Deep LPF	3D-LUT	Proposed Method	Original Images
5	29	39	36	33	37	36	44	89
7	22	37	32	30	35	35	42	89

## Data Availability

The datasets used in this work are publicly available. The DIV2K dataset can be accessed at https://data.vision.ee.ethz.ch/cvl/DIV2K/ (accessed on 18 March 2026). The MPEG Compact Descriptors for Visual Search (CDVS) dataset is available at https://mpeg.chiariglione.org/standards/mpeg-7/compact-descriptors-visual-search/n14960-working-draft-2-cdvs-conformance-testing.html (accessed on 18 March 2026). The Oxford Buildings dataset can be accessed at https://www.robots.ox.ac.uk/~vgg/data/oxbuildings/ (accessed on 18 March 2026), and the Paris Buildings dataset at https://www.robots.ox.ac.uk/~vgg/data/parisbuildings/ (accessed on 18 March 2026).

## Abbreviations

The following abbreviations are used in this manuscript:

DoG	Difference of Gaussian
SIFT	Scale-Invariant Feature Transform
PSNR	Peak Signal-to-Noise Ratio
CNN	Convolutional Neural Network
SE	Squeeze-and-Excitation
ReLU	Rectified Linear Unit
LUT	Lookup Table
HDR	High Dynamic Range
LPF	Local Parametric Filters
CDVS	Compact Descriptors for Visual Search
DoF	Degree of Freedom
